# Cognitive potential of children and adolescents with CHARGE syndrome and deafblindness

**DOI:** 10.1186/s13023-024-03222-w

**Published:** 2024-06-11

**Authors:** Lynn Skei, Sigmund Skei, Timothy Hartshorne, Nils Inge Landrø

**Affiliations:** 1 Signo Resource Centre, Sandefjord, Norway; 2 Department of Psychology, University of Oslo, Oslo, Norway; 3 Department of Health and Care, County Governor Vestfold & Telemark, Tønsberg, Norway; 4 Department of Psychology, Central Michigan University, Mt Pleasant, MI USA

**Keywords:** CHARGE, Congenital deafblindness, Cognitive potential, Atypical development, Cognitive assessment

## Abstract

**Background:**

The present study aimed to test the hypothesis stating that the cognitive potential of individuals with deafblindness is equal to those without a deafblind condition, an assumption that until now has been empirically unsubstantiated within the field of deafblindness.

**Methods:**

To explore the assumption, 15 children and adolescents with CHARGE underwent cognitive assessment with WISC-V using a sequential two-level assessment design. The 1st level involved standardized test conditions. The 2nd level was designed as a continuation of the performances obtained from the 1st level and involved accommodations to compensate for sensory motor impairment. Statistical procedures involved the sample as a whole and when divided into two subgroups: (i) participants with CHARGE without deafblindness; (ii) participants with CHARGE and deafblindness using the 1st level scores as base line.

**Results:**

Although results showed significantly lower scores in the deafblind subgroup with standardized procedures, they approximated the others after accommodating for their sensory deficits. This positive increase proved significant.

**Conclusion:**

Findings supported the assumption of equal cognitive potential of individuals with and without deafblindness. Results indicated that the children and adolescents with deafblindness had most effect of the accommodations, enabling them to approximate the results of the subgroup without deafblindness. These gains were attributed enhanced accessibility endorsed by the accommodations and represented the participants latent cognitive dispositions only realized under certain circumstances.

## Background

As soon as the pregnancy is known to the parents and other family members, anticipation and worry about the child's progress and potential begin to mould. These are soon shared by local health care, social services, and educational systems. If a child deviates from the expected developmental trajectory, parents and professionals soon intervene medically and educationally to get the child back on track. However, some children may, from the very beginning of life, follow an atypical path filled with unexpected halts, setbacks, and sudden growth spurts. Such deviations from the expected often causes confusion and uncertainty in parents’ and professionals’ perception of the child's developmental potential. Without knowledge of the range of potentials, a path of ambivalence usually follows (e.g., what means can realise the child's latent potential). Thus, the likelihood for the child to reach more advanced stages of competency increases with more knowledge of *what might be possible* and vice versa.

The combined sensory-motor impairment of children and adolescents with CHARGE makes them vulnerable to atypical progress [1, 2]. In addition to such propensity, many uncertainties exist regarding the neuropsychological potential, causing special educational hesitancy [3]. Fortunately, the prevailing attitude among researchers and clinicians working within the field of deafblindness and rare disorders is that the affected child or adolescent has an unlocked cognitive potential [4–6]. Because of methodological issues connected to research on rare diseases, this belief has proven challenging to substantiate scientifically. The present study aims to close a small part of this knowledge gap.

While learning opportunities accumulate seemingly effortlessly for neurotypical children [7], this is rarely the case for individuals with combined sensory impairments. Being born with impaired hearing and vision constrain type and frequency of opportunities of learning by negatively impacting environmental accessibility. Accordingly, "the net effect dramatically affects the child's overall development of cognition and communication" ([8], p. 214). These circumstances are the reality for most children with CHARGE [4, 9, 10].

CHARGE (OMIM 214800) is a rare genetic condition characterised by a wide phenotype heterogeneity [1, 2]. Its genotype, mutations in the gene encoding the ATP-dependent chromatin remodelers Chromodomain Helicase DNA-binding protein-7 (CHD7) (OMIM 608892) [11], is considered the leading cause of major and minor characteristics (https://www.sense.org.uk/). Already from the embryonic stage, multileveled changes emerge and propagate (e.g., neuro crest cells, cranial nerves, sensory systems, brain, and cognition). Clinical manifestations of these cascades include distinct sensorimotor features, permeative cognitive challenges, and behavioural idiosyncrasies [12]. The high incidence of combined anomalies within the auditory [13] and visual pathways [14] significantly impact their psychomotoric progress and outcome. Between 50 and 70% of individuals with CHARGE have severe degrees of sensory challenges corresponding to a deafblind condition [9, 15], defined as:

"a condition, in which the degree of hearing and visual impairment [makes it impossible for the individual] to use one sense to fully compensate for the impairment of the other" [16].

Deafblindness is not considered a medical diagnosis delineated in the diagnostic manuals [17, 18] but a functional description. Consequently, individuals become *identified* with deafblindness rather than diagnosed.

Because of the many secondary difficulties, including limitations in social life, mobility, and access to information, deafblindness is recognised as a distinct disability [16, 19]. A combined condition (i.e., CHARGE *and* deafblindness) gives even more significant challenges in daily living. These characteristics make children and adolescents with CHARGE prone to present atypical patterns of progress from the postnatal stage and onwards.

Notably, deafblindness rarely implies total loss of both primary senses. The affected individuals most often have some residual hearing and vision, enabling them to perceive fragments of their immediate environment. For example, despite being functionally blind, tunnel vision allows identification and understanding of distinct parts of social contexts. Likewise, the residual hearing may give access to parts of the acoustic spectrum essential when seeking knowledge of the outer world. Combined with other sources of sensorimotor information (e.g., vibrations from the surroundings, movement of the body posture, and changes of airflow on their skin), the individual with deafblindness can obtain contextual knowledge from recognition of social cues and patterns.

Literature addressing individuals with CHARGE underlines their inherent compensatory capacity and latent developmental potential in functions and ability level [10, 20–22]. However, like other rare disorders methodological issues impede quantitative behavioural studies [23, 24]. In addition to the sample size challenges, most psychometric measures rely on operative primary senses, which excludes many individuals with combined auditory and visual impairments since enhancing accessibility impact the validity of findings. Accordingly, few quantitative studies involving performance-based assessment exist [24].

Adjustments of the standardised assessment procedure seem likely when conducting quantitative performance-based research of individuals with CHARGE, which raise validity concerns. Whereas norm-referenced assessment involves minor changes and considered equivalent with standardized methodology [25], accommodations aims to increase the sensorimotor accessibility of individuals with disabilities enabling them to demonstrate their inherent competency [26, 27]. Opposite to modifications [28], which involve more comprehensive changes compromising the construct validity [29, 30], accommodated test results may undergo norm-based evaluation insofar as the purpose is optimize development and not medical diagnosis (e.g., diagnosis of intellectual disability) [17, 31].

### Present study

By investigating the cognitive functioning of 15 children and adolescents with CHARGE using a two-level assessment design with the Wechsler Intelligence Scale for Children, Fifth Edition (WISC-V) [32], this study aimed to test the hypothesis stating that individuals with deafblindness have equal cognitive potentials as those without deafblindness.

## Methods

### Sample

The sample of the present study’s originated from a cross-sectional population study comprising nearly all individuals known with CHARGE in Norway (*N* = 35) [33]. This primary population (*N* = 35) demonstrated statistical representativity on several intellectual parameters (i.e., normally distributed), such as the overall measure of intellectual abilities. Basing the selection on age requirements of WISC-V (i.e., age interval [6.0, 16.11]) resulted in a sub-sample of 15 children and adolescent, which equalled most school aged children with CHARGE in Norway. Accordingly, assuming continued representativity, the same parameters should remain valid.

The gender ratio of the 15 children and adolescents was 7 to 8 (7 boys), between the ages of 7.6 and 16.11 (*M* = 12.7).

The degree of auditory deficits varied among the participants: 3 had a slight auditory impairment, 5 had moderate, and 7 had severe or profound, along with visual challenges: 2 presented average vision, 5 slight impairments, 1 moderate, and 7 severe.

Four participants used sign language while the others communicated by sign supported speech. Notable, Norwegian sign language has the same legal and educational status as Norwegian speech.

The sample was divided into two subgroups. The first subgroup consisted of 8 participants without deafblindness with a mean age of 12.3 years. The second subgroup included 7 participants with mean age of 13.2 years presenting more extensive combined sensory impairments corresponding to a deafblind condition. In Norway, a multidisciplinary team connected to *the National Advisory Unit on Deafblindness, Department of the University Hospital og Northern Norway* conduct the evaluation and final identification of deafblindness. Accordingly, all the participants had been evaluated by this unit as a part of their ordinary medical follow-up.

### Design

**Fig. 1:**
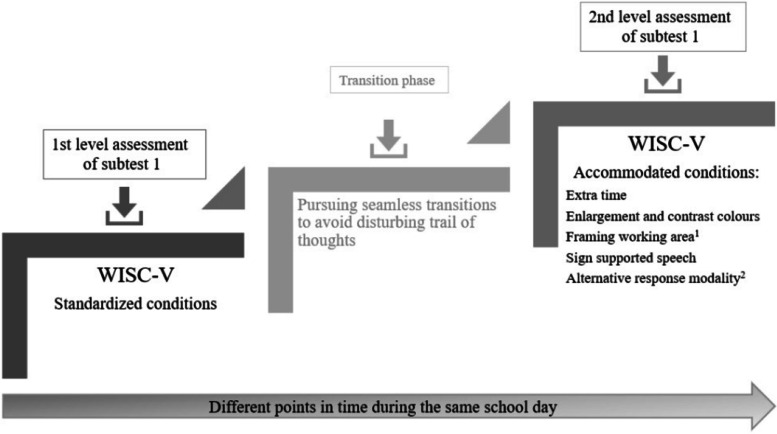
Graphic illustration of the study design

This study applied a two-level assessment approach (Fig. 1), in which the 1st level refers to a standardized administration of WISC-V without deviating from the test protocol [32].

The 2nd level assessment was a continuation of the 1st level using the stop criteria from the level 1 as the starting point for level 2. The main goal of this level was to increase accessibility by providing compensation (i.e., accommodation) for the participants combined sensory impairments, and founded on the principle of augmentative and alternative communication support (AAC) (https://isaac-online.org/english/what-is-aac/).

While the participants standard scores on WISC-V provided a baseline for comparative analyses, the norm data served as control group. The participants degree of hearing and visual impairments was defined as independent variables. A gap between a participant’s test score and expected score (i.e., relevant age norms on WISC-V) should imply the effect of intervening factor(s).

### Cognitive measures and arithmetic’s

The subtests *Block design*, *Similarities*, *Digit span*, *Matrix reasoning*, *Coding*, *Figure weights* and *Vocabulary* of WISC-V [32] estimated the participants cognitive functioning on two levels. A *Full-Scale Index* referred to their performances after completing the 1st level assessment following standardized procedures. A *Global Cognitive Estimate* designated an overall score estimated after completing the 2nd level, which included gains in scores due to the provided accommodations. An *Estimated Cognitive Difference* represented the difference in scores between the two levels.

The test norms of WISC-V evaluated participants’ performances from both levels.

### Implementation

The first author, a clinical neuropsychologist, conducted all the anamnestic interviews, preparation, and execution of testing.

Every participant was assigned up to 5 days to undergo all testing. Each day was divided into two working sessions, one morning and one midday, of no more than 45 min. The child’s teacher, a fluent signer, attended all the test sessions.

The 1st level assessment followed standardized procedure, i.e., each participant went through sequentially the items on every subtest until reaching the stop criteria. The assessment proceeded at the same subtest to the 2nd level by enforcing a seamless transition. This involved introduction of different accommodations, starting at the three recently failed test items from the 1st level.

The 4 participants who used sign language as their primary language needed instructions and responses translated (i.e., Norwegian speech to Norwegian Sign Language). Because different grammatical rules apply in Norwegian Sign language compared to Norwegian speech, the wording of the test instructions and verbal subtests had to be changed as illustrated below:

- Standardized Question: What is the similarity between Red and Green?
- Sign Language Connotation: Red! Green! Similar, how?
- Standardized Question: Shy. What does shy mean?
- Sign Language Connotation:Shy! Means what?
- Standardized Question:Why should we avoid throwing garbage in the nature?
- Sign Language Connotation:Garbage in nature, not good! Why?

Each item underwent analysis to evaluate if the change of wording influenced the content and intent. In case of uncertainties the item was excluded for all the participants.

### Test accommodations

All the participants received accommodations regardless of their sensory motor impairment. Building on the original Wechsler material and reports from parents’ and teachers’, the accommodations’ final design addressed challenges within hearing, vison, and psychomotor tempo [34]. Furthermore, the categories delineated by Sireci and O’Riordan served as a guideline (i.e., *Presentation*, *Response*, and *Timing*) [27]. The *Presentation* category included enlargement of test material, application of contrasts and bold print, and framing the working area by Velcro boards (i.e., on the school desk). It also involved repeating instructions, sign supported speech and tactile communication.

The *Response* category involved alternative augmentative communication beyond speech and pointing, e.g., Velcro patches, crossing out, and tracing to indicate answers.

The *Timing* category consisted of extra time for completing each test item disregarding the time-limits indicated in the original test protocol.

All 15 received language support (i.e., visual and tactile signs) during the 2nd level assessments.

The visual accommodations included enlargement of print and supplication of contrasts to separate essential information from irrelevant visual noise (e.g., framing of the task at hand with black, brighter colors).

Extra time for task completion served to compensate for the participants’ tempo challenges.

Due to the crucial importance for the present study’s credibility, the discussion includes validations of the provided accommodations.

### Statistical analyses

Tests of normality of findings were overall found satisfactory for further statistical investigations. The skewness of the Full-Scale Index (0.97) and Global Cognitive Estimate (1.01) appeared moderate and left-skewed. Their kurtosis equalled 1.23 and 1.17, respectively, indicating a more light-tailed distribution than a Gaussian distribution. The Shapiro–Wilk test turned out significant for both variables (Full-Scale Index: *p* = 0.04; Global Cognitive Estimate: *p* = 0.03). The effect size (Cohen’s d) turned out small as expected due to the small sample but given this paper's purpose not precluding further statistical analyses.

Pearson correlations estimated the strength of the associations between the dependent (i.e., Full-Scale Index, Global Cognitive Estimate, and Estimated Cognitive Difference) and independent variables (i.e., age, gender, degree of sensory impairment, including deafblindness). In contrast, paired sample t-tests estimated their difference.

Data analyses used SPSS software (version 29.0) with significance level *p* < 0.05.

## Results

### Qualitative data

Accumulation of fatigue from one day of testing to the next applied for all the participants. Due to their need of frequent breaks and premature endings of planned test sessions, the amount of time required to complete the assessment increased. Most (*n* = 12) needed 4 to 5 days to finish both levels. The participants (*n* = 3) who finished after three test sessions also demonstrated the best performance and least exhaustion.

Two participants with deafblindness scored at floor level on the 1st level assessment, but both obtained quantifiable results at level 2. The type and degree of health issues causing frequent school absenteeism and barriers to learn when attending separated them from the others.

The most potent accommodations (i.e., the most impact on the 2nd level performances) included extra time, enlargement of prints and answer sheets, and communicating through preferred modalities (e.g., sign and tactile supported speech).

For the youngest participants, allowing alternative response styles more in accordance with those applied in school greatly impacted their compliance and test scores.

The subtests *Matrix reasoning*, *Coding*, and *Figure weights* appeared most accommodation sensitive by presenting the most significant positive change in scores when subtracting 2nd level performances from 1st level performances. Whereas the accommodations had minimal effect on *Block design* and *Similarities* performances, the subtest *Digit span* demonstrated a mixed picture. Although accommodations improved the *Forward memory* score, this was not the case for the *Backward condition*.

### Quantitative data

**Fig. 2:**
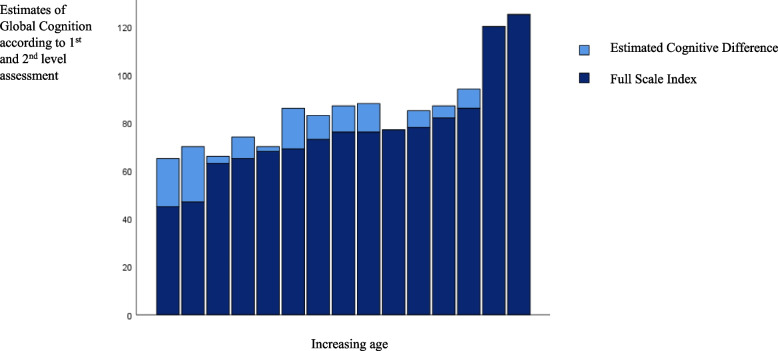
The participants’ test performances after the 1st and 2nd level assessments

**Fig. 3:**
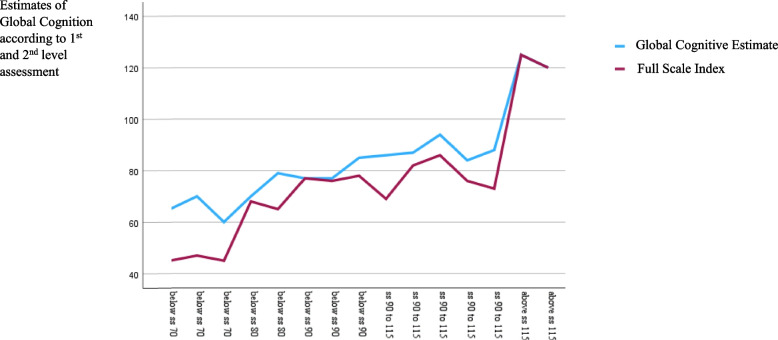
Illustration of the differences in the participants’ performances after the 1st and 2.^nd^ assessments (*N* = 15)

Performances (*N* = 15) from the level 1 assessments (i.e., standardized) yielded a mean Full-Scale Index of 78.5 (*SD* = 17.4). The mean Global Cognitive Estimate obtained after level 2 assessments (i.e., adaptations to increase access) equalled 87.5 (*SD* = 22.8). The difference, 9.0, turned out significant (*t*(14) = -5.5, *p* < 0.001) (Fig. 2). A line diagram revealed the variance in scores between level 1 and level 2 performances according to cognitive intervals (Fig. 3).

### Associations between predictors and differences in performance level

The Estimated Cognitive Difference (*N* = 15) correlated significantly with the participants' degree of auditory impairment (*r* = 0.6, *p* = 0.03) and deafblindness (*r* = 0.6, *p* = 0.01) but not the degree of visual impairment (*r* = 0.2, *p* = 0.41). The positive effects of the accommodations appeared most prominent for the youngest participants (Fig. 2) and those with the lowest performance scores (Fig. 3).

### Comparison of performances of participants with and without deafblindness

The difference between the outcomes from the two assessment conditions emerged as significant (*F*(15) = 6.9, *p* = 0.02).

**Fig. 4:**
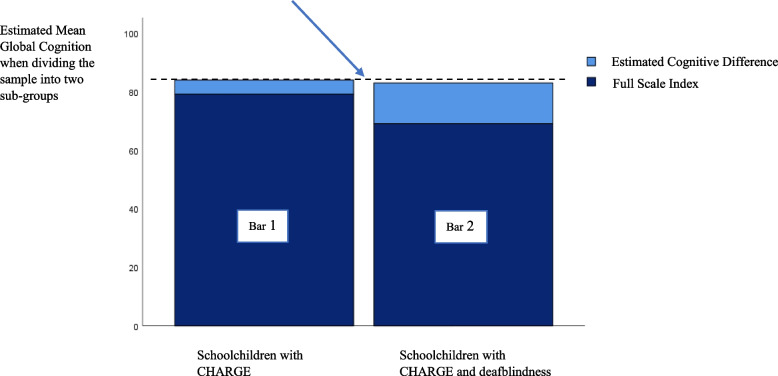
Graphic presentation of the estimated mean performances from 1st and 2nd level assessments when comparing the subgroup with and without deafblindness

Figure 4 gives a graphical presentation of the quantification of the performances of participants without deafblindness (*n* = 7) (first bar) and participants with deafblindness (*n* = 8) (second bar). Each bar comprised two estimates: results from the standardized test procedure (dark blue area) and the relative increase in performance level after giving the participants increased auditory and visual access during testing (light blue area).

A comparison of the total height of each bar demonstrates approximately identical performance levels (*M* ≈ 84) (Fig. 4, black dotted line). This equilibrium (Fig. 4, blue arrow) illustrates the relative gains of accommodations as a function of deafblindness.

## Discussion

Before proceeding with the discussion of findings, an issue of concern requires special attention. The test scores obtained from the 2nd level assessment (i.e., performances after providing accommodation) cannot serve as diagnostic parameters, such as estimates in the diagnosis of intellectual disability [17, 18]. Instead, the increase in scores between the two levels provides a measure of the effect of enhanced accessibility on test performances. The accommodations that bring about these positive changes are primarily tools to guide future interventions and optimise functions and abilities.

Building on three premises, the two-level design in the present study found quantitative support for one of the prevailing assumptions in the field of deafblindness in need of scientific substantiation [24], i.e., that individuals with deafblindness have equal cognitive potential as those without deafblindness when accounting sufficiently for their sensory deficits [4].

The study's first premise (I) states the necessity to identify a significant increase in scores when comparing performance from the 1st and 2nd level assessment and with relative certainty represent real effects (e.g., not measurement errors).

The statistical analyses demonstrated a significant change in scores after introducing the accommodations. All the participants underwent the same two-level assessment procedure by the same clinician; test items included during the 2nd level were mainly new (i.e., only repetition of the stop-criteria), and evaluation of each participant's 1st and 2nd level scores based on the same set of age norms [32]. Accordingly, the increase in scores could not ascribed to practice effects and test–retest errors, a concern when a research population serve as their own control [35]. Furthermore, each participant served as their own control, which constrained the number of measurement errors. The probability of identifying real effects from any experimental manipulation, such as the provided accommodations, increased.

The second premise (II) holds that any increase in the performance levels should mainly be an effect of enhanced accessibility rather than facilitated test conditions (i.e., no influence of the difficulty level). Verification of this premise lies in the two-level design and the nature of the accommodations. The main change between the 1st and 2nd level assessments included the provided accommodations, which aimed to enhance sensorimotor accessibility by providing the participants verbal and visual support (e.g., sign-supported speech, enlargement of prints and answer sheets, and extra time for sensory processing). Furthermore, all the accommodations originated from the WISC-V test material without changing or incorporating new tasks or more beneficial instructions. The subtest's construct validity should remain relatively unaffected ([34]). The positive increase between 1st and 2nd levels (premise I) is likely an effect of the provided accommodations.

The subtests targeting visual analysis and synthesis, and psychomotoric tempo (i.e., *Matrix reasoning*, *Coding*, and *Figure weights*) presented the highest positive accommodation effects (e.g., the extra time and enlargement). In contrast, test items connected to verbal abstraction remained relatively unchanged regardless of overall test results. By other CHARGE studies (e.g., [33, 34]) and research on congenital hearing impairment and language development (e.g., [36, 37]), these findings may indicate that verbal abstraction is particularly challenging for individuals with CHARGE. Test items related to working memory (i.e., *Digit span backwards*) appeared insensitive to the accommodations, which mainly involved signing and repeating the number sequence one extra time. However, the effect was apparent when applied to the *Forward* condition, which mainly tap attention. Other CHARGE studies have also found a robust and operative working memory capacity [33, 34].

Considering the provided accommodations as beneficial (i.e., made the test items easier) would imply that the gain in performance level represented an overestimation of abilities rather than an estimate of the participant's cognitive potential. Delineation of the complex comorbidity in CHARGE, affecting all reference levels, can contradict such objection. Being born with CHARGE entails a range of somatic complications and sensorimotor challenges affecting the child's mobility, cognition, adaptive behaviour, and psychoemotional functioning (e.g., [38, 39]). While other children obtain large amounts of knowledge relatively effortlessly through implicit and experiential learning, the features of CHARGE constrain these learning opportunities [3, 40, 41]. Learning and knowledge generally rest on the environment's ability to enhance focus and access to one part of reality before turning to the next. Such explicate learning is both time-consuming and mentally demanding.

Furthermore, while the accommodations intended to increase accessibility, they can also induce new domain-related challenges and even enhance the mental workloads [42]. For example, while their neurotypical peers quickly scan and locate visual targets, individuals with CHARGE provided enlarged alternatives must inspect and process a much larger visual field and activate working memory functions to a greater degree. Similarly, providing tactile support can compensate for visual deficits yet require more complex sensory integration. In the present study, the participants presented sudden exhaustion or fatigue-like symptoms during cognitive challenges. Despite increased accessibility, the accommodations also cause an enhanced toll on energy expenditure [43–45].

The study's third premise (III) holds that an increase in performance level mainly represents a cognitive potential when controlling for the effect of sensory impairment. This statement rests on theoretical principles within dynamic assessment theory (i.e., a known scholastic procedure for exploration of the student accomplishment during supported learning) [45–48], which operates with the notions actual (i.e., what they can achieve on their own) and potential learning abilities (i.e., how much they can achieve with support from a more knowledgeable). Accordingly, the participants' 1st level performances may signify an actual learning ability by defining the provided accommodations as a support mechanism. Since these abilities appear as latent dispositions only realised in certain circumstances (i.e., when provided means to enhance accessibility), they represent a potential outcome. Furthermore, the estimates in the current study originated from an intelligence scale (WISC-V) rather than a scholastic setting (i.e., potential learning abilities), so cognitive potential becomes a more appropriate term.

With the affirmation of the three premises above, the discussion can return to the initial hypothesis. Findings from the 1st level assessment obtained by the two subgroups demonstrated that the scores of children and adolescents with deafblindness were significantly lower than the scores of those without deafblindness. Results connected to the 2nd level assessments revealed almost equal performances between the two subgroups, implying that the children and adolescents with deafblindness gained the most from the provided sensory compensations. Their performance gains enabled them to catch up and reach approximately the same ability level as the participants without deafblindness. Because the realisation of these skills depended on an intervention (e.g., the accommodations provided), the gain in performance levels of either subgroup remains a potential until provided the proper support.

Based on the previously affirmed premises and findings from comparative analyses involving the two subgroups, the present study supported the hypothesis of equal cognitive potential between individuals with and without deafblindness.

### Limitations

Even though it is a natural consequence in all research addressing low-frequent conditions, small sample sizes always represent a limitation by influencing statistical power. Furthermore, because no national register of individuals born with CHARGE exists in Norway, the actual prevalence remains to be discovered. However, since Norway is a small country some indication of administrative prevalence exists [33]. While tests of normality give some guidance regarding the samples' representativity, uncertainties still prevail in the inferences drawn from findings.

## Conclusion

The sequential two-level assessment approach in the present study demonstrated that increasing the participants sensory accessibility had positive effect on their test performances. The participants cognitive potential turned out independent of deafblindness or degree of sensory impairment. Whereas outcomes from the adapted assessment cannot automatically serve as valid estimates in assessing and diagnosing intellectual disabilities, discovering an association between achievement level, and auditory and visual compensation can have several implications for the learning and development of children with CHARGE. Recommendations would, for example, highlight the importance of ascertaining environmental accessibility from birth. The findings also highlight the likelihood of latent learning capacity of children and adolescents with CHARGE, in which realization depend on the environments ability to free them from handling compensatory processes. This competency can then be redirected and utilized on crucial areas of learning, which for in CHARGE is language and communication.

## Data Availability

The dataset of this study consisted of three graphical presentations: Figure 2 is a graphic presentation of the differences in the participants performances level after the level 1 and level 2 assessment (before and after test adaptations) for the entire sample (i.e., undivided). Statistical analyses subtracted the participants results obtained from to the first level of assessment, from the results obtained by the second level assessment. Figure 3 is just another graphic presentation of the findings in Fig. 1. Figure 4 is a graphic presentation of the estimated mean performances from level 1 and level 2 assessments when comparing the subgroup without deafblindness with the subgroup with deafblindness. Figure 1 is only a methodological illustration and contain no data. Because of the dataset consists of a small sample with a rare condition dataset are not openly available but can be provided by the Signo Resource Centre on request: post.ssk@signo.no. https://www.signo.no/virksomheter/signo-kompetansesenter/ The sample of this study is extracted from another published: 10.1080/00207454.2023.2240485

## Abbreviations

AAC: Augmentative and Alternative Communication
ATP: Adenosine Tri Phosphate
CHARGE: Coloboma of the eye, Heart defects, Atresia of the choanae, Restriction of growth and development, and Ear abnormalities and deafness
WISC-V: Wechsler Intelligence Scale for Children, 5th edition
